# Internet use in old age predicts smaller cognitive decline only in men

**DOI:** 10.1038/s41598-020-65846-9

**Published:** 2020-06-02

**Authors:** Andreas Ihle, Daphne Bavelier, Jürgen Maurer, Michel Oris, Matthias Kliegel

**Affiliations:** 1 Department of Psychology, University of Geneva, Geneva, Switzerland; 2 Center for the Interdisciplinary Study of Gerontology and Vulnerability, University of Geneva, Geneva, Switzerland; 3 Swiss National Centre of Competence in Research LIVES – Overcoming vulnerability: Life course perspectives, Lausanne and Geneva, Switzerland; 4 Department of Economics, University of Lausanne, Lausanne, Switzerland

**Keywords:** Human behaviour, Geriatrics, Alzheimer's disease

## Abstract

Internet use provides cognitive stimulation and thereby may contribute to the accumulation of cognitive reserve that is proposed to be instrumental for maintaining cognitive health in ageing. As the first study so far, we examined possible gender differences in the relationship between Internet use and subsequent cognitive decline over six years assessed through changes in Trail Making Test (TMT) accomplishment time in 897 older adults. Latent change score modelling (taking into account baseline cognitive level, chronic diseases, age, and central contributions to cognitive reserve through education, profession, and leisure engagement) revealed a significant interaction of frequency of Internet use and gender. More frequent Internet use in the first wave of data collection significantly predicted a smaller subsequent augmentation in TMT accomplishment time (i.e., a smaller subsequent cognitive decline) only in men, but not in women. In conclusion, frequent Internet use may contribute to the accumulation of cognitive reserve. The gender difference noted highlights an advantage for males. While this finding could be interpreted as gender-specific, it may be that the Internet activities males engage in differ from those of females, calling for a fine-grained investigation of Internet-based activities in future studies.

## Introduction

Due to the demographic changes, with more and more adults attaining older ages, but, at the same time, also an increase of people suffering from cognitive impairments, the preservation of cognitive health in old age constitutes one of the major challenges in this century^1–3^. In this regard, the cognitive reserve concept hypothesises that cognitive stimulation throughout the lifespan, including education, profession, and leisure engagement, augments the individual’s reserve capacity that is proposed to be instrumental for maintaining cognitive health in ageing^4,5^. For instance, it is assumed that inter-individual differences in the cognitive processes and involved neural networks account for inter-individual differences in the capacity to sustain pathology and age-related decline^6–11^. Empirically, the assumptions of the cognitive reserve concept regarding the contributions to the accumulation of cognitive reserve throughout the lifespan have been confirmed by a large body of correlational evidence. For example, higher educational attainment in early life, cognitively demanding professions in working life, and greater leisure engagement in midlife and old age have been found to be related to better performance in a broad variety of cognitive tests assessing e.g. memory, processing speed, and attentional control in old age^12–19^. Moreover, these cognitive reserve contributors have been found to be associated with a lower risk of developing dementia and later age at dementia onset^20–24^.

In this regard, Internet use may represent a cognitively challenging leisure activity and as such provide cognitive stimulation and contribute to the accumulation of cognitive reserve. For instance, disentangling the reciprocal longitudinal relationships between Internet use and cognitive functioning over a 2-year period using a cross-lagged panel analysis, Kamin and Lang^25^ found that Internet use had a greater impact on cognitive functioning than vice versa. Further empirical evidence suggests that frequent Internet use may help to reduce cognitive decline in older adults^26,27^. This evidence dovetails with similar findings showing that frequent computer use in general is related to better cognitive functioning in old age^28,29^. Yet, to the best of our knowledge, existing research on these issues did not explicitly focus on investigating potential gender differences in the relationship between Internet use and cognitive decline^30^. However, considering gender differences in this context may be potentially important. This is because one of the most robust results in the literature on technology use in general is that technology use differs widely between gender^30–33^. For instance, it has been well documented in younger populations that frequency of technology use (including the Internet), the type of technology devices used, and the context and behaviour of use differ by gender^31–33^. Importantly, such differences in use have been associated to different cognitive impact. For example, a recent meta-analysis in younger samples showed that cognitively demanding action video games elicit larger effects on cognitive functioning than social simulation or puzzle video games^34^, a concern as the action game genre is dominated by male players, while social simulation and puzzle games more readily attract female players. Interestingly, experimental studies have established that action video game play can be used to reduce gender differences in spatial cognition^35^. In contrast, in the aging and technology literature, the specific consequences for aging outcomes of gender differences in technology use remain quite mixed^36–43^. Accordingly, in their recent review, Hunsaker and Hargittai^30^ stress the urgent need of studies better representing the diversity of older populations, especially taking up a nuanced perspective on the relationship between gender and Internet use among older adults.

With the present longitudinal study, we aimed to target this important knowledge gap. Specifically, we examined the relationship between frequency of Internet use in the first wave of data collection and subsequent cognitive decline over six years assessed through changes in Trail Making Test (TMT) accomplishment time. We investigated whether this longitudinal relationship differed between women and men, taking into account baseline cognitive level, chronic diseases, age, and central contributions to cognitive reserve through education, profession, and leisure engagement.

## Results

### Descriptive statistics

Mean accomplishment time in TMT A was 55.23 seconds (*SD* = 24.40) in W1 and 56.03 seconds (*SD* = 24.37) in W2. Mean accomplishment time in TMT B was 115.13 seconds (*SD* = 44.80) in W1 and 108.90 seconds (*SD* = 45.40) in W2. Comparing both waves, there was no difference in accomplishment time in TMT A nor TMT B on average (*p*s > 0.145).

With respect to first-order correlations, more frequent Internet use in W1 was significantly related to a faster accomplishment of TMT A in W2 (but not W1) as well as TMT B in both waves. Higher education was significantly related to a faster accomplishment of TMT B (but not TMT A) in both waves. Higher cognitive demand of the first job was significantly related to a faster accomplishment of TMT A in W2 (but not W1) as well as TMT B in both waves. Higher cognitive demand of the last job was significantly related to a faster accomplishment of TMT A in both waves as well as TMT B in W2 (but not W1). A larger number of leisure activities in W1 was significantly related to a faster accomplishment of both TMT A and TMT B in both waves. A larger number of chronic diseases in W1 was significantly related to a slower accomplishment of TMT A in W2 (but not W1) as well as TMT B in both waves. Older age was significantly related to a slower accomplishment of both TMT A and TMT B in both waves. Women accomplished the TMT A in W2 significantly faster than men. Otherwise, TMT accomplishment time did not differ by gender. By contrast, women used the Internet significantly less frequently than men (see Table 1 for the full correlation matrix).

**Table 1:** Full correlation matrix of measures.

Variable	1	2	3	4	5	6	7	8	9	10	11
1. TMT A accomplishment time (W1)	–										
2. TMT A accomplishment time (W2)	0.38***	–									
3. TMT B accomplishment time (W1)	0.55***	0.38***	–								
4. TMT B accomplishment time (W2)	0.35***	0.63***	0.49***	–							
5. Frequency of Internet use (W1)	−0.06 ns	−0.20***	−0.21***	−0.21***	–						
6. Education (0 = low; 1 = high)	−0.08 ns	−0.05 ns	−0.10*	−0.10*	0.27***	–					
7. Cognitive demand first job (0 = low; 1 = high)	−0.08 ns	−0.12***	−0.14***	−0.23***	0.16***	0.39***	–				
8. Cognitive demand last job (0 = low; 1 = high)	−0.09*	−0.08*	−0.07 ns	−0.17***	0.22***	0.36***	0.51***	–			
9. Number of leisure activities (W1)	−0.20***	−0.28***	−0.18***	−0.25***	0.18***	0.09**	0.09**	0.08*	–		
10. Number of chronic diseases (W1)	0.04 ns	0.15***	0.08*	0.15**	−0.08*	0.00 ns	0.01 ns	−0.06 ns	−0.15***	–	
11. Age (W1)	0.19***	0.34***	0.27***	0.40***	−0.27***	0.01 ns	−0.08*	0.01 ns	−0.33***	0.15***	–
12. Gender (0 = men; 1 = women)	0.00 ns	−0.07*	−0.01 ns	0.03 ns	−0.21***	−0.06 ns	0.19***	−0.03 ns	0.00 ns	0.06 ns	−0.06 ns

*Note*: First-order correlations between accomplishment time in TMT A and TMT B in Wave 1 (W1) and Wave 2 (W2), frequency of Internet use in W1, education, cognitive demand of the first and of the last job, the number of leisure activities in W1, the number of chronic diseases in W1, age in W1, and gender.

***p < 0.001; **p < 0.01; *p < 0.05; ns = non-significant, *p* > 0.05.

### Latent change score modelling

We adopted a latent change score modelling approach previously reported by Ihle *et al*.^44,45^ using the R package lavaan^46^. Compared to a simple regression model with change scores as dependent variable, the latent change score modelling approach has the advantage of extracting measurement-error variance^47^, which is especially crucial when modelling intra-individual changes^45,48,49^. This is because individual change scores as dependent variable in a simple regression model are much less reliable than the changes modelled as a latent variable by means of a latent change score model^45,48,49^. In general, latent variables are measurement-error-free, thus with perfect reliability^47^. For these reasons, the present latent change score modelling approach is preferable over the alternative approach of analysing individual change scores in TMT parts A and B separately as dependent variables with simple regression models^45,48,49^.

**Figure 1:**
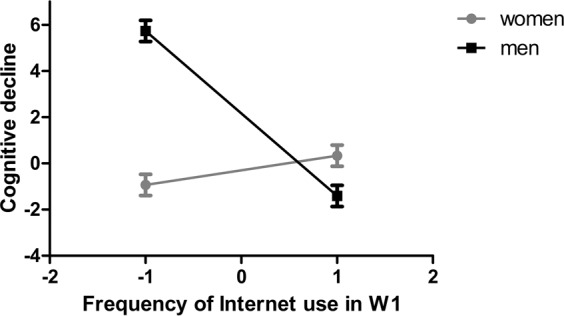
Illustration of the interaction of frequency of Internet use with gender on latent change. Estimated mean augmentation in Trail Making Test (TMT) accomplishment time (i.e., cognitive decline in seconds) from the first wave (W1) to the second wave of data collection at a low and a high frequency of Internet use in the first wave (i.e., −1 and +1 *SD*, respectively) as a function of gender. Bars represent standard errors. Note that the dots at a low and a high frequency of Internet use (i.e., −1 and +1 *SD*, respectively) are for descriptive purposes only. For analyses we did not artificially dichotomise frequency of Internet use (into low vs. high) and instead analysed it as a continuous score. Reprinted from Neuropsychologia, 121, Ihle *et al*.^44^, Copyright (2018), with permission from Elsevier.

The latent change score model provided a good statistical account of the data (*χ²* = 36.58, *df* = 20, *p* = 0.013, *CFI* > 0.99, *IFI* > 0.99, *RMSEA* = 0.03, *SRMR* = 0.02). In this model, the covariates predicted 27.6% of the variance in the latent change in TMT accomplishment time. Specifically, more frequent Internet use in W1 significantly predicted a smaller subsequent augmentation in TMT accomplishment time (i.e., a smaller subsequent cognitive decline, *b* = −3.57, *p* = 0.002, 95% *CI*: −6.06 to −1.43, corresponding standardised estimate *β* = −0.22). Gender (*b* = −2.46, *p* = 0.084, 95% *CI*: −5.09 to 0.34, corresponding *β* = −0.08), education (*b* = 1.50, *p* = 0.336, 95% *CI*: −1.39 to 4.72, corresponding *β* = 0.05), cognitive demand of the first job (*b* = −1.82, *p* = 0.276, 95% *CI*: −4.86 to 1.51, corresponding *β* = −0.05), and cognitive demand of the last job (*b* = −1.38, *p* = 0.533, 95% *CI*: −5.49 to 3.12, corresponding *β* = −0.03) did not predict change in TMT accomplishment time. A larger number of leisure activities in W1 significantly predicted a smaller subsequent augmentation in TMT accomplishment time (i.e., a smaller subsequent cognitive decline, *b* = −2.27, *p* = 0.001, 95% *CI*: −3.67 to −0.84, corresponding *β* = −0.14). A larger number of chronic diseases in W1 (*b* = 1.21, *p* = 0.017, 95% *CI*: 0.16 to 2.17, corresponding *β* = 0.12) and older age in W1 (*b* = 4.15, *p* < 0.001, 95% *CI*: 2.35 to 5.70, corresponding *β* = 0.26) significantly predicted a larger subsequent augmentation in TMT accomplishment time (i.e., steeper subsequent cognitive decline). There was a significant interaction of frequency of Internet use in W1 with gender (*b* = 4.21, *p* = 0.013, 95% *CI*: 1.05 to 7.76, corresponding *β* = 0.18). Specifically, more frequent Internet use in W1 significantly predicted a smaller subsequent augmentation in TMT accomplishment time (i.e., a smaller subsequent cognitive decline) only in men (*b* = −3.57, *p* = 0.002, 95% *CI*: −6.06 to −1.43, corresponding *β* = −0.22), but not in women (*b* = 0.63, *p* = 0.618, 95% *CI*: −1.93 to 2.98, corresponding *β* = 0.04; cf. Figure 1).

Regarding the correlations between the latent cognitive factor in W1 and the W1 covariates, more frequent Internet use significantly correlated with a faster TMT accomplishment (i.e., better cognitive performance level, *r* = −0.22, *p* < 0.001, 95% *CI*: −5.51 to −2.12 for corresponding raw estimate *b* of −3.78). Gender (*r* = 0.01, *p* = 0.885, 95% *CI*: −0.67 to 0.77 for corresponding *b* of 0.05) did not correlate with TMT accomplishment time. Higher education (*r* = −0.14, *p* = 0.002, 95% *CI*: −1.89 to −0.43 for corresponding *b* of −1.18), higher cognitive demand of the first job (*r* = −0.15, *p* < 0.001, 95% *CI*: −1.99 to −0.50 for corresponding *b* of −1.24), higher cognitive demand of the last job (*r* = −0.10, *p* = 0.039, 95% *CI*: −1.27 to −0.04 for corresponding *b* of −0.63), and a larger number of leisure activities in W1 (*r* = −0.27, *p* < 0.001, 95% *CI*: −6.14 to −3.26 for corresponding *b* of −4.62) significantly correlated with a faster TMT accomplishment (i.e., better cognitive performance level). A larger number of chronic diseases in W1 (*r* = 0.10, *p* = 0.010, 95% *CI*: 0.55 to 4.68 for corresponding *b* of 2.67) and older age (*r* = 0.32, *p* < 0.001, 95% *CI*: 4.19 to 7.08 for corresponding *b* of 5.54) significantly correlated with a slower TMT accomplishment (i.e., lower cognitive performance level).

### Supplemental analysis

To explore whether our findings were robust over the broad age range in our study sample, in an additional analysis we tested whether the pattern observed was moderated by age. For this purpose, we additionally entered a three-way interaction of frequency of Internet use in W1 * gender * age in the aforementioned latent change score model. In this model, the pattern remained largely unchanged: More frequent Internet use in W1 still significantly predicted a smaller subsequent augmentation in TMT accomplishment time (i.e., a smaller subsequent cognitive decline, *b* = −3.52, *p* = 0.003, 95% *CI*: −5.75 to −1.13, corresponding *β* = −0.22). Again, there was a significant interaction of frequency of Internet use in W1 with gender (*b* = 4.33, *p* = 0.014, 95% *CI*: 0.75 to 7.89, corresponding *β* = 0.18). Specifically, more frequent Internet use in W1 significantly predicted a smaller subsequent augmentation in TMT accomplishment time (i.e., a smaller subsequent cognitive decline) only in men (*b* = −3.52, *p* = 0.003, 95% *CI*: −5.75 to −1.13, corresponding *β* = −0.22), but not in women (*b* = 0.70, *p* = 0.584, 95% *CI*: −1.73 to 3.16, corresponding *β* = 0.04). Importantly, there was no significant three-way interaction of frequency of Internet use in W1 * gender * age on latent change in TMT accomplishment time (*b* = 1.47, *p* = 0.277, 95% *CI*: −1.32 to 4.18, corresponding *β* = 0.06), indicating that the previously observed two-way interaction of frequency of Internet use in W1 with gender on latent change in TMT accomplishment time could not be attributed to a specific age cohort, but rather applied across age cohorts.

## Discussion

The present longitudinal study examined the relationship between frequency of Internet use and subsequent cognitive decline over six years assessed through changes in TMT accomplishment time. We explored whether this longitudinal relationship differed between women and men. Latent change score modelling taking into account baseline cognitive level, chronic diseases, age, and central contributions to cognitive reserve through education, profession, and leisure engagement showed that more frequent Internet use in the first wave of data collection predicted a smaller subsequent augmentation in TMT accomplishment time (i.e., a smaller subsequent cognitive decline). In general, this result confirms prior evidence suggesting that frequent Internet use may help to reduce cognitive decline in old age^26,27^ and that frequent computer use in general is related to better cognitive functioning in old age^28,29^. Importantly, testing for potential gender differences in the present study, we found differential patterns between women and men with regard to the relationship between more frequent Internet use and smaller subsequent cognitive decline. Specifically, we found that more frequent Internet use in the first wave of data collection predicted a smaller subsequent augmentation in TMT accomplishment time (i.e., a smaller subsequent cognitive decline) only in men, but not in women.

The present findings have several implications. First, our observation of a relationship between frequency of Internet use and smaller subsequent cognitive decline over and above central contributions to cognitive reserve through education, profession, and leisure engagement suggests that frequent Internet use may directly contribute to the accumulation of cognitive reserve. This finding dovetails with several studies suggesting that besides the important role of early-life academic performance^21,22^, stimulating activities in later life additionally contribute to the accumulation of cognitive reserve over the lifespan^15,50,51^.

Second, as we observed the relationship between more frequent Internet use and a smaller subsequent augmentation in TMT accomplishment time only in men, but not in women, we show that the relationship between frequency of Internet use and smaller subsequent cognitive decline may differ in important ways for different socio-demographic groups. Specifically, in the present data, we did not observe gender differences in education or the number of leisure activities, but only in frequency of Internet use (with women using the Internet less frequently than men). With respect to the latter observation, importantly, we controlled for the interdependencies of gender with frequency of Internet use and all other variables in our analyses. Therefore, gender-specific relationships between frequency of Internet use and smaller subsequent cognitive decline observed in the present study cannot simply be explained by gender differences in frequency of Internet use per se.

The present study documents, using a 6-year longitudinal design, a robust relationship between frequency of Internet use and smaller subsequent cognitive decline over and above central contributions to cognitive reserve through education, profession, and leisure engagement. We acknowledge that the correlative study design does not allow causal inferences. Thus, we cannot fully disentangle a potentially protective effect of frequent Internet use on subsequent cognitive decline from alternative explanations related to the potential self-selection of older adults with better cognitive abilities to become more frequent users of new technologies like the Internet. Yet, importantly, given that our analyses are based on cognitive longitudinal change scores and took into account baseline cognitive level in the first wave of data collection (when frequency of Internet use was assessed), it is less probable that the observed relationship between more frequent Internet use and a smaller subsequent cognitive decline is due to individuals who reduced Internet use because of cognitive decline. This rationale dovetails with recent empirical research disentangling the reciprocal longitudinal relationships between Internet use and cognitive functioning over a 2-year period using a cross-lagged panel analysis and demonstrating that Internet use has a greater impact on cognitive functioning than vice versa^25^. The view that cognitive stimulation protects against cognitive decline is also supported by experimental research such as the intervention regime included in the seminal World Wide Fingers Trial^52,53^. In this trial, a computerised process-based cognitive training was applied that is modelled after well-known laboratory-based working memory, episodic memory, and cognitive control training interventions^54^. Such experimental designs will be key to draw causal conclusions regarding the potential effects of cognitive stimulation on subsequent cognitive ageing. We note, however, that cognitive reserve research focuses on everyday-life activities that are somewhat different from participating in a study evaluating standardised cognitive training programmes. As cognitive reserve research aims at examining long-term relationships between everyday-life cognitive activities (such as education, profession, or as in the present case frequency of Internet use) on cognitive decline over many years, the field has had so far to mainly rely on correlative study designs.

A strength of the present study is the use of latent change score modelling as we aim to capture intra-individual changes in cognitive performance. Importantly, we controlled for several possible confounding factors. First, our modelling controlled for *education, past occupations, leisure activities that have been associated with the build-up of cognitive reserve*. One might also argue that the role of frequency of Internet use for subsequent cognitive changes may be confounded with health issues. Yet, importantly, we controlled for the interdependencies of chronic diseases with frequency of Internet use and all other variables. It is therefore rather unlikely that present observations regarding the role of frequency of Internet use simply reflect individual differences in health status.

Cognitive abilities were assessed through TMT parts A and B in the present study, and our analyses focused on the latent change *in TMT accomplishment over a 6-year period*. One could argue that comparing both waves, there was no difference in accomplishment time in TMT A nor in TMT B on average. Importantly, the present study focused on explaining inter-individual differences regarding intra-individual change (not change on average). Despite non-significant average change in ageing, there are in general large inter-individual differences in intra-individual change over time^55–58^. This was the case in our sample and present results show that there was a sufficient amount of inter-individual differences in intra-individual change in TMT accomplishment time to be able to detect differential relationship patterns with respect to frequency of Internet use and gender.

Given our use of only the TMT as cognitive evaluation, we could not investigate whether the observed pattern of results is specific for the TMT (that involves cognitive flexibility and processing speed and that was therefore used as a sensitive measure of inter-individual differences in intra-individual cognitive change^44,59^) or whether there may be differences depending on the cognitive abilities studied. In this respect, prior empirical evidence suggests that Internet and computer use in general may predict performance outcomes in a larger variety of cognitive domains also including memory and executive functioning^27,29^. In addition, one could argue that both TMT parts A and B include a motor component and that therefore it may be possible that motor function played a role in the observed relationships. Importantly, given that we focused on inter-individual differences (not absolute values), if the majority of individuals within the range of healthy development show similar changes with respect to motor functioning, these changes should only marginally affect inter-individual differences in intra-individual change in the outcome (i.e., TMT accomplishment time) since they would not affect the rank order of individuals. Moreover, given that we controlled for a variety of chronic diseases (that may also be indicative of preclinical motor problems), present analyses may to some extent be robust with respect to the confounding role of motor function also beyond the range of healthy development. Yet, as outlined, we acknowledge that future research will have to further scrutinise these issues by administering a broader variety of cognitive tests.

We report a robust gender difference in the relationship between frequency of Internet use and smaller subsequent cognitive decline. In particular, more frequent Internet use in the first wave of data assessment predicted a smaller subsequent cognitive decline only in men but not women. Importantly, this gender difference was robust across age cohort differences, i.e., a broad range of age cohorts spanning more than 30 years, as well as several other variables in which women and men in different age groups may differ, including education, past occupations, leisure activities, and diseases. *Therefore, gender-specific relationships between frequency of Internet use and cognitive decline observed in the present study cannot simply be explained by gender differences in frequency of Internet use per se*.

One could argue that gender is not a static category but a social role that varies with the life course and each new cohort. If so, gender differences with regard to Internet use patterns found in prior studies^60–63^ will tend to replicate in subsequent cohorts only to the extent that gender in these cohorts share similar social roles. We review below the conceptual and empirical indications that the social roles in our study cohort are largely similar to those in the cohorts for which gender differences in Internet use patterns have been documented^60–63^. First, conceptually, social gender roles are established mostly via primary socialisation processes already early in life^64^. In this respect it is also worth to consider that empirical evidence from our group showed that when the participants of our study cohort were children and young adults, they have been socialised in a Swiss society that was quite conservative in general, and especially as far as the gender roles were concerned. All the channels of socialisation (families, schools, youth movements, churches, etc.) promoted a role of women as mother and spouse^65^. Second, besides this most crucial primary socialisation in early life, empirical evidence further showed that although the shift in subjective perceptions of social gender roles towards being less conservative over the last 30 years in Switzerland due to secondary socialisations during the life course, importantly, the social practices were changing across time and generations to a much lower extent: Women were still much more than men engaged in social activities in the private space and the managers of social relationships, especially family relationships and friendship^66,67^ (for further discussions on these issues see e.g.^68,69^).

In this respect, the relationship between Internet use and cognitive outcomes may not be universal, but rather be determined, for example, by the exact Internet activity patterns of women and men. One potential explanation for the gender difference observed may be that (beyond frequency of use per se) women and men may differ in their respective motivations and exact patterns of Internet use, which, in turn, may affect its potential influence on cognitive reserve and cognitive functioning in later life. Previous evidence suggested that older men seem to use the Internet on a wider range of devices and for a broader range of activities than women. For example, older men’s use of the Internet seems dominated by information and research purposes, while older women seem to mainly use the Internet for communicating with family and friends^60–63^. On first glance, it may sound counterintuitive to expect gender differences in cognitive outcomes in this context because cognitive reserve research showed that social activities such as the maintenance of social relationships are an important source of cognitive stimulation in later life, which are beneficial for cognitive performance in old age^23^. Yet, most importantly, cognitive reserve research also showed that cognitive activities seem to be more strongly associated with reduced decline in global cognition, compared to social activities^18^, and that social activities were more strongly associated with a broad spectrum of cognitive abilities if those social activities also involved substantial cognitive demands^18,24^.

These are certainly the early days when it comes to understanding the source of gender differences in Internet use and how they may affect cognitive reserve accumulation. *We recognise that our study constitutes only a first foray as our technology use questions were rather limited, having been designed in the late 2000. Since then, a growing number of studies indicates that the features of the technology itself (such as the type of media, its content, the delivery interface, the nature of the interaction, and the context of use) are crucial to answer the key open question of how in detail individuals may profit or suffer from their technological environments in everyday life*^70–72^*. In this regard, our results highlight the need of going beyond simply analyses of Internet use frequency, but rather develop more detailed questionnaires distinguishing passive viewing from active use, cognitively challenging use from habitual activities, and probing the richness of social interactions during technology use, to cite a few. This expansion will also help to further scrutinise the role of technology in the broader context of aging and lifespan development*^42^.

By identifying a robust gender difference, the present study highlights the importance for future research to characterise potential differences in the cognitive complexity and demand of Internet use patterns between women and men and ask if these may be the sources in gender differences in the relationship between Internet use and cognitive outcomes.

To summarise, the present findings are important for the field of aging and technology in general. Detailed longitudinal research in the field of aging and technology is sparse, results on the relationship between technology and their outcomes in later life are mixed, and therefore it is a hot topic in the field to better understand how in detail and under which conditions technology can have positive effects on aging^42,43^. Specifically, our study highlights the need of considering inter-individual difference characteristics, specifically gender, in the relation between technology (such as Internet use) and adaptive aging outcomes.

## Methods

### Participants

Data come from two waves of the Vivre-Leben-Vivere (VLV) survey^15,44,73,74^. Respondents were first interviewed during 2011 (Wave 1; W1) using face-to-face computer-assisted personal interviewing (CAPI) and paper-pencil questionnaires. The main stratified random sample of 3080 participants in W1 based on administrative records from five Swiss cantons (Basel, Bern, Geneva, Ticino, and Valais) with stratification by age, sex, and canton^75^. A subsample of 1059 participants from four cantons (Basel, Bern, Geneva, and Valais) was interviewed again during 2017 (Wave 2; W2). Present analyses were based on 897 participants with data on TMT parts A and B as these were needed to measure the outcome variables of interest in the present study. The mean age of these respondents in W1 was 74.33 years (*SD* = 6.50, range 64–96).

Reflecting the longitudinal study design, our sample only contained survivors. Compared to all participants initially tested in W1, the participants retained in the present study were younger (*M* = 74.33 years in W1, *SD* = 6.50) than the individuals who were lost at follow-up in W2 (*M* = 80.00 years in W1, *SD* = 8.60; *p* < 0.001). Importantly though, our sample still contained a considerable proportion of respondents aged 85 years and older in W2 (24.5% in W2 among the participants who were analysed in the present study; in comparison, 25.7% in W1 among the participants initially tested in W1). Present participants were also more frequently using the Internet in W1 (*M* = 1.79, *SD* = 1.30; see below for further scale descriptions) than the individuals who were lost at follow-up in W2 (*M* = 1.06, *SD* = 1.34; *p* < 0.001). However, a comparable proportion of respondents reported using the Internet only infrequently (less than once per week) in W1 (9.8% among the participants who were analysed in the present study; in comparison, 7.4% among the participants initially tested in W1). The fraction of male vs. female participants did not differ significantly between our analytical sample and individuals who were lost at follow-up in W2 (51.4% vs 51.9% men, *p* = 0.811). However, the retained participants (43.2% with low education, 30.0% with a low cognitive demand of the first job, and 15.9% with a low cognitive demand of the last job) differed somewhat from the individuals who were lost at follow-up in W2 (58.9% with low education, 46.0% with a low cognitive demand of the first job, and 31.1% with a low cognitive demand of the last job) with regard to their education and cognitive demands of their jobs (*p*s < 0.001). However, in our analytical sample we still had data on a considerable proportion of respondents from low education and job categories (for comparison: in the overall sample initially tested in W1 there were 53.6% with low education, 39.9% with a low cognitive demand of the first job, and 24.2% with a low cognitive demand of the last job). The participants retained in the present study also had pursued slightly more leisure activities in W1 (*M* = 9.94, *SD* = 2.82) than the individuals who were lost at follow-up in W2 (*M* = 7.31 in W1, *SD* = 3.44; *p* < 0.001) but our sample still contained a considerable proportion of respondents in the lower range of leisure activity participation (23.6% individuals with seven or fewer activities among the participants who were analysed in the present study; in comparison, 42.6% among the participants initially tested in W1). Moreover, the participants retained in the present study had suffered from slightly fewer chronic diseases in W1 (*M* = 1.90, *SD* = 1.56) than the individuals who were lost at follow-up in W2 (*M* = 2.49 in W1, *SD* = 2.10; *p* < 0.001). However, we still had a considerable proportion of respondents with four or more chronic diseases (29.7% among the participants who were analysed in the present study; in comparison, 21.1% among the participants initially tested in W1), suggesting sufficient variance in multimorbidity in the study sample. Thus, systematic attrition did not eliminate entire population groups of interest (see^76^ for comparable retention patterns over six years in the Longitudinal Aging Study Amsterdam; see^77^ for a similar follow-up of participants over six years in the Victoria Longitudinal Study; see^78^ for a similar follow-up of participants over four years in the Survey of Health, Ageing and Retirement in Europe).

All participants gave their written informed consent for inclusion in the study before participating. The present study was conducted in accordance with the Declaration of Helsinki, and the study protocol had been approved by the ethics commission of the Faculty of Psychology and Social Sciences of the University of Geneva (project identification codes: CE_FPSE_14.10.2010 and CE_FPSE_05.04.2017).

## Materials

### Trail making test accomplishment time

We administered in both waves the Trail Making Test part A (TMT A^79^). After one exercise trail (connecting the numbers from 1 to 8), participants had to connect the numbers from 1 to 25 as fast as possible and without error in ascending order. The TMT A accomplishment time was the time in seconds needed to correctly connect the 25 numbers.

In addition, we administered in both waves the Trail Making Test part B (TMT B^79^). After one exercise trail (connecting 1-A-2-B-3-C-4-D), participants had to connect the numbers 1 to 13 in ascending order and the letters A to L in alphabetic order while alternating between numbers and letters (i.e., 1-A-2-B-3-C… 12-L-13) as fast as possible and without error. The TMT B accomplishment time was the time in seconds needed to correctly connect the 25 numbers / letters.

### Frequency of internet use

We asked participants in W1 how frequently they were usually using the Internet, based on a five-point Likert-type scale ranging from 0 (“never”) to 4 (“every day, more than three hours per day”).

### Education

We asked participants in W1 to indicate their highest educational level attained. For analyses, we distinguished low versus high education as follows. Low educational attainment comprised primary and inferior secondary school levels and apprenticeship graduation, both leading mainly to blue collar and/or unskilled jobs. Higher (advanced) educational attainment represented superior secondary school level, technical college or superior vocational college, and university degree, typically leading to white collar jobs^80^.

### Cognitive demand of job

We asked participants in W1 to indicate their first profession after completing education as well as their last profession before retirement. For analyses, we distinguished low versus high cognitive demand of job regarding individuals’ first profession and the last profession practiced as follows. Lower cognitive demands comprised blue collar or unskilled jobs such as factory work, plumbing, carpentry, farming, etc. Higher cognitive demands represented white collar jobs such as teacher, clerical work, lawyer, medical practice, etc. (see^12,81^ for similar ratings reflecting the degree of intellectual involvement at work as marker of cognitive reserve).

### Leisure activities

We also asked participants in W1 their leisure activities such as going to the cinema, going to conferences, journeys, artistic activities, table games, and municipality activities. For our analyses, we calculated the overall number of leisure activities reported by participants in W1.

### Chronic diseases

We interviewed participants in W1 regarding the chronic diseases they suffered from, such as heart diseases of ischemic or organic pathogenesis, primary arrhythmias, pulmonary heart diseases, hypertension, and peripheral vascular diseases. For analyses, we summed up the overall number of chronic diseases participants suffered from in W1 as a global indicator of individuals’ multimorbidity^82,83^.

### Statistical analyses

**Figure 2:**
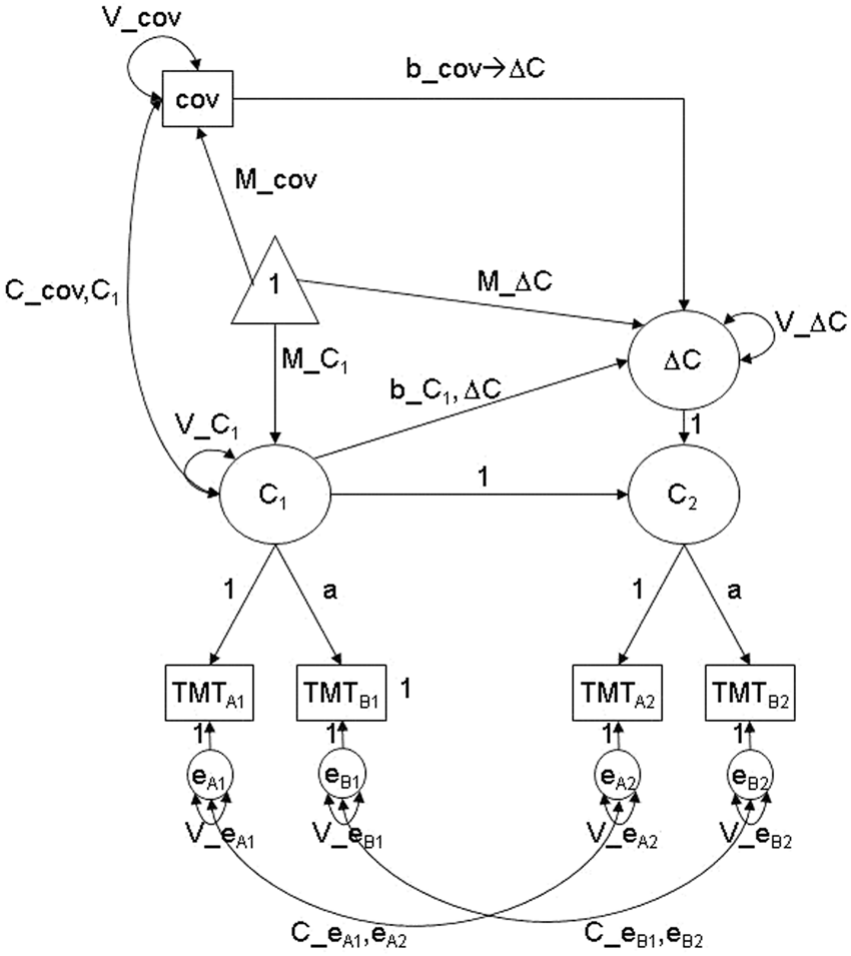
Specification of the tested latent change score model. C_1_ and C_2_ represent the latent cognitive factors of Trail Making Test (TMT) accomplishment time in Wave 1 (W1; constructed from scores in TMT parts A and B in W1) and Wave 2 (W2; constructed from scores in TMT parts A and B in W2), respectively. ΔC represents the latent change variable regarding change in TMT accomplishment time from W1 to W2. Note that for clarity purposes the illustration is simplified. We enforced strong metric invariance on the factor loadings, with intercepts of all indicators being fixed to zero to assure that the same cognitive factor was assessed at both waves. For simplification purposes, arrows from the triangle to the observed indicator variables (TMT A and B) that would indicate that intercepts of all indicators being fixed to zero are not displayed. cov represents all W1 covariates that predicted latent change and were correlated to the latent cognitive factor in W1: Frequency of Internet use in W1, gender, education, cognitive demand of the first and of the last job, the number of leisure activities in W1, the number of chronic diseases in W1, age in W1, and the interaction of frequency of Internet use in W1 with gender (including interrelations of all covariates, which are not displayed here for a better overview)^44^.

The specification of our latent change score model is illustrated in Fig. 2. Specifically, we modelled latent cognitive factors of TMT accomplishment time in W1 (constructed from scores in TMT parts A and B in W1) and W2 (constructed from scores in TMT parts A and B in W2) as well as a latent change variable regarding change in TMT accomplishment time from W1 to W2. We enforced strong metric invariance on the factor loadings, with intercepts of all indicators being fixed to zero to assure that the same cognitive factor was assessed at both waves. We included several W1 covariates that predicted latent change and were correlated to the latent cognitive factor in W1: Frequency of Internet use in W1, gender, education, cognitive demand of the first and of the last job, the number of leisure activities in W1, the number of chronic diseases in W1, age in W1, and the interaction of frequency of Internet use in W1 with gender. We also included interrelations of all covariates (taking the dependencies among them into account).

For model estimation, we used full information maximum likelihood. We calculated bootstrapped standard errors (based on 1000 bootstrap draws). We additionally inspected the bootstrapped 95% confidence intervals (*CI*s). We evaluated model fit as follows: Given that with large study samples the *χ²* test often indicates a significant deviation of the model matrix from the covariance matrix despite good model fit^84^ we inspected several additional fit indices. Specifically, we used the following criteria: Comparative Fit Index (good models: *CFI* > 0.95), Incremental Fit Index (good models: *IFI* > 0.95), Root Mean Square Error of Approximation (good models: *RMSEA* < 0.06), and Standardised Root Mean Square Residual (good models: *SRMR* < 0.08)^84^. Frequency of Internet use, the number of leisure activities, the number of chronic diseases, and age were analysed as continuous variables. We standardised frequency of Internet use, the number of leisure activities, and age so that the reported raw estimates (*b*) can be interpreted in terms of *SD*s. We did not standardise the number of chronic diseases because it allowed interpreting the reported raw estimates in terms of relations “for each additional chronic disease”^44^. We did not standardise accomplishment time in TMT A or TMT B so that the reported raw estimates can be interpreted in seconds^44^.

## Supplementary information

Dataset 1.

## Data Availability

Data are available online as supplemental material.
